# Gettysburg Battle Field

**Published:** 1888-07

**Authors:** 


### GETTYSBURG BATTLE FIELD.

The re-union of the veterans of the Federal and Confederate armies who fought in opposition upon the bloody field of Gettysburg on the memorable days of July 1, 2, and 3, 1863, which is to take place on that field on the 25th anniversary of the battle, will be the first great assemblage of its kind known to history, and could hardly transpire in any other country than ours. The war was brought about by the Southern States to dissolve the national compact and perpetuate the slave system which prevailed among them, whilst the leading motive of the North was to preserve the union from disruption and dismemberment. The victory of the North was not over a people so much as over an institution which from its nature, was inimical to free government and natural rights. There never had been and never could be harmony between the two sections with this bone of contention in the foreground and the crisis that precipitated an appeal to arms could only be decided after a long and bloody contest.

That of Gettysburg may be said to be the decisive battle of the war. Had the Confederate forces then prevailed, there is no telling what might have been the consequences, but it is certain that such an overthrow to the defenders of the Union would have appealed strongly to the sympathizers with the South, and prolonged the war indefinitely. From this unexpected defeat, Lee's Army never fully recovered, and after a series of battles the following year, extending over a period of nearly two months of continuous fighting, beginning in the Wilderness and ending with the occupation of Petersburg, his army was once more concentrated in and about Richmond, to lay down their arms almost at the outset of the spring campaign. The surrender was followed in rapid succession by that of all the armies and strong-holds of the Confederacy.

From first to last the rank and file of the two armies were in no proper sense enemies. As citizens of a common country they had been arrayed upon opposing sides upon a single issue and when that issue was decided their hostile attitude ceased, it is to be hoped, for all time. Now they meet with open palms and mutual good fellowship strengthened, rather than embittered by the sacrifices of the past.

Two events of the Gettysburg struggle will be ever memorable in its history. They were the forced march of the sixth Corps to relieve their brothers in arms, almost worn out in the first two days of battle, and the terrible charge and repulse of Pickett's division of Longstreet's Corps, upon our batteries, in the red tide of battle, on the third day ; that tremendous anset which was to decide the wage of battle.

This historic field is now a National Cemetary, consecrated to the patriotic dead by the Martyr President who stood upon the watch tower, throughout the darkest days of the Republic unswayed by passion and undismayed by the rush of the tempest and guided the destinies of the nation. Amid the multiplicity of monuments that dot the landscape, sacred alike to the living and the dead, one is lacking. It should be erected as a peace offering by all the States, to stand forever as a token of reconciliation, re-union, and the solidaritè of this Republic of States. Let it rise from its foundation in the order of states from the first to the last, each state contributing its allotted space, so far as practicable, out of its native product in granite trap or marble.

Such an emblem would be at once a warming and a bond ever admonishing, ever revered.

> "The boast of heraldry, the pomp of power,
>   And all that beauty, all that wealth e'er gave,
> Await alike the inevitable hour,
>   The paths of glory lead but tn the grave."

